# Imaging spectrum of sporadic cerebral amyloid angiopathy: multifaceted features of a single pathological condition

**DOI:** 10.1007/s13244-014-0312-x

**Published:** 2014-02-12

**Authors:** Keita Sakurai, Aya M. Tokumaru, Tomoya Nakatsuka, Shigeo Murayama, Shin Hasebe, Etsuko Imabayashi, Kazutomi Kanemaru, Masaki Takao, Hiroyuki Hatsuta, Kenji Ishii, Yuko Saito, Yuta Shibamoto, Noriyuki Matsukawa, Emiko Chikui, Hitoshi Terada

**Affiliations:** 1 Department of Diagnostic Radiology, Tokyo Metropolitan Medical Centre of Gerontology, 35-2 Sakaecho, Itabashi-ku, Tokyo, 173-0015 Japan; 2 Department of Radiology, Toho University Sakura Medical Centre, Sakura, Japan; 3 Department of Neuropathology (the Brain Bank for Aging Research), Tokyo Metropolitan Geriatric Hospital, Tokyo Metropolitan Geriatric Hospital and Institute of Gerontology, Tokyo, Japan; 4 Department of Neurology, Tokyo Metropolitan Geriatric Hospital, Tokyo, Japan; 5 Positron Medical Centre, Tokyo Metropolitan Institute of Gerontology, Tokyo, Japan; 6 Department of Pathology and Laboratory Medicine, National Centre for Neurology and Psychiatry Hospital, Tokyo, Japan; 7 Department of Radiology, Nagoya City University Graduate School of Medical Sciences, Nagoya, Japan; 8 Department of Neurology and Neuroscience, Nagoya City University Graduate School of Medical Sciences, Nagoya, Japan; 9 Department of Neurosurgery, Tokyo Metropolitan Geriatric Hospital, Tokyo, Japan

**Keywords:** Cerebral amyloid angiopathy, Imaging, Subarachnoid haemorrhage, Microbleed, Superficial siderosis, CAA-related inflammation

## Abstract

**Objectives:**

Sporadic cerebral amyloid angiopathy (CAA) is common cause of cerebrovascular disorders that predominantly affect elderly patients. When symptomatic, cortical-subcortical intracerebral haemorrhage (ICH) in the elderly is the most well-known manifestation of CAA. Furthermore, the clinical presentation varies from a sudden neurological deficit to seizures, transient symptoms and acute progressive cognitive decline. Despite its clinical importance, this multifaceted nature poses a diagnostic challenge for radiologists. The aims of this study were to expound the characteristics of neuroimaging modalities, which cover a wide spectrum of CAA-related imaging findings, and to review the various abnormal findings for which CAA could be responsible.

**Conclusions:**

Radiologically, in addition to typical ICH, CAA leads to various types of abnormal findings, including microbleed, subarachnoid haemorrhage, superficial siderosis, microinfarction, reversible oedema, and irreversible leukoaraiosis. Taking into consideration the clinical importance of CAA-related disorders such as haemorrhagic risks and treatable oedema, it is necessary for radiologists to understand the wide spectrum of CAA-related imaging findings.

***Teaching Points*:**

• *To describe the characteristics of imaging modalities and findings of CAA-related disorders.*

• *MRI, especially gradient echo sequences, provides the useful information of CAA-related haemosiderin depositions.*

• *To understand the wide spectrum of CAA-related neuroimaging and clinical features is important.*

## Introduction

Sporadic cerebral amyloid angiopathy (CAA) is a common small vessel disease of the brain, characterised by the progressive deposition of amyloid-β (Aβ) protein in the walls of small- to medium-sized arteries (up to about 2 mm in diameter), arterioles and capillaries in the cerebral cortex and overlying leptomeninges, preferentially affecting occipital regions for unclear reasons. In contrast to the amyloid plaques found in Alzheimer disease (AD), which are predominantly composed of the 42-amino-acid-residue fragment, the vascular amyloid in CAA is mostly composed of the more soluble, 40-amino-acid fragment, which suggests different pathophysiological mechanisms for pathological deposition [1]. Impairment in one or more elimination mechanisms may result in the accumulation of Aβ in the walls of small- and medium-sized leptomeningeal and cortical blood vessels. Upon autopsy, CAA may be found more commonly in women than in men. The incidence of CAA, like AD, is strongly age-dependent. Although found at autopsy in only 33 % of 60–70 year olds, the prevalence of age-related CAA increases to 75 % among those older than 90 years [2]. Despite its high prevalence, CAA remains an underestimated cause of cerebrovascular disease, both clinically and at imaging, in part because many patients are asymptomatic. When symptomatic, intracerebral haemorrhage (ICH) in the elderly is the most well-known manifestation. Furthermore, the clinical presentation varies from a sudden neurological deficit to seizures, transient symptoms and cognitive decline, including acute progressive dementia. However, these symptoms are not specific and are often not readily associated with CAA.

Radiologically, in addition to typical acute cortical-subcortical ICH, CAA leads to various types of abnormal findings, including chronic ICHs, microbleed (MB), subarachnoid haemorrhage (SAH), superficial siderosis (SS), microinfarction, reversible oedema and irreversible leukoaraiosis (Table 1) [3]. Taking into consideration the clinical importance of CAA-related haemorrhagic risks in the setting of antiplatelet, anticoagulation and thrombolysis therapies, as well as treatable oedema [3–6], it is necessary for radiologists to understand the wide spectrum of CAA-related imaging findings.

**Table 1:** CAA-related abnormal imaging findings

Disease	Imaging findings	Recommended neuroimaging modality
ICH	Haematoma with distinctive cortical-subcortical distribution generally sparing the deep white matter and basal ganglia and brainstem	CT and MRI **MRI**; additional depiction of chronic haemosiderin depositions and MBs
MBs	Small round hypointense foci on MRI	MRI, especially susceptibility-weighted image
SAH	Supratentorial sulcal high attenuation/intensity, most frequently depicted around the precentral gyrus	CT and MRI **MRI**; additional depiction of MB and SS
SS	Hypointensity along the supratentorial cerebral sulcus on MRI	MRI, especially susceptibility-weighted image
CAA-related inflammation	Large confluent asymmetric abnormal attenuation/intensity mainly in the subcortical WM	CT and MRI **MRI**; additional evaluation of vasogenic oedema and other findings such as MB and SS
Leukoaraiosis	Low attenuation on CT and high intensity on FLAIR and T2W MRI mainly in the deep WM with sparing of the subcortical U fibres	CT and MRI MRI; depiction of leukoaraiosis clearer than CT
Microinfarction	Small ovoid or round high intensity of the subcortical and cortex on diffusion-weighted image	MRI, especially diffusion-weighted image

*CAA* cerebral amyloid angiopathy, *CT* computed tomography, *FLAIR* fluid-attenuated inversion recovery, *ICH* intracerebral haemorrhage, *MRI* magnetic resonance imaging, *MB* microbleed, *SAH* subarachnoid haemorrhage, *SS* superficial siderosis, *T2W* T2-weighted, *WM* white matter

The aims of this study were the following: to expound the characteristics of neuroimaging modalities, including computed tomography (CT), magnetic resonance imaging (MRI) and positron emission tomography (PET), which cover a wide spectrum of CAA-related imaging findings, and to review the various abnormal findings for which CAA could be responsible. The recognition of wide-spectrum imaging findings can be useful for radiologists not only to raise the possibility of CAA but also to precisely comprehend the pathophysiology of CAA and management to improve the prognosis.

## Neuroimaging modalities: critical roles in the diagnosis of CAA

### CT

CT is the initial screening modality for patients with various symptoms, especially acute neurological deficits or transient ischaemic attack-like symptoms, which can allow rapid establishment of the presence or absence of ICHs and SAHs. CT can provide crucial information regarding the characteristics of these haemorrhagic conditions, including volume, shape and distribution. Additional CT angiography with intravenous contrast media is useful to exclude other pathological conditions (e.g. aneurysms, arteriovenous malformation, fistula and venous thrombosis) that could cause similar haemorrhagic complications.

On CT scan, cortical-subcortical ICHs without a history of hypertension and sulcal SAHs without a history of head trauma can be the findings suggestive of CAA. However, it is difficult to evaluate other findings, such as MBs and SS, which support the diagnosis of CAA. The disadvantage of CT is lower contrast resolution than MRI, which can depict acute cerebral infarctions, MBs and white matter lesions more clearly. In other words, CAA cannot be diagnosed by CT alone, but requires MRI sequences sensitive to susceptibility effects.

### MRI

The important point of MRI in the diagnosis of CAA is to perform the proper sequences to cover a wide spectrum of CAA-related abnormal findings including not only haemorrhages but also oedemas and infarctions. Therefore, the standard imaging protocol should include at least the gradient-echo (GRE), fluid-attenuated inversion recovery (FLAIR) and diffusion-weighted imaging (DWI). The standard MRI protocol is shown in Table 2.

**Table 2:** Standard MRI protocol for the diagnosis of CAA

Sequence	Expected role for the diagnosis
Minimum required protocol	
T2*-weighted image	Depiction of haemosiderin depositions suggestive of chronic ICHs, MBs and SS
Fluid-attenuated inversion recovery image	Depiction of acute and subacute SAHs, and white matter signal changes
Diffusion-weighted image	Depiction of acute microinfarction Evaluation of vasogenic oedema due to CAA-related inflammation
Optional sequence for the diagnosis of CAA	
Susceptibility-weighted image	Depiction of haemosiderin depositions, clearer than T2*-weighted image
Additional sequences for differential diagnosis	
T1-weighted image	Depiction of T1 shortening due to methaemoglobin and melanin
Contrast-enhanced T1-weighted image	Differentiation between haemorrhagic tumours and other lesions
Magnetic resonance angiogram	Evaluation of vascular disorders such as vasculitis

*CAA* cerebral amyloid angiopathy, *ICH* intracerebral haemorrhage, *MB* microbleed, *MRI* magnetic resonance imaging, *SAH* subarachnoid haemorrhage, *SS* superficial siderosis

**Fig. 1:**
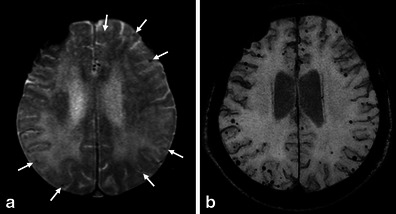
Multiple MBs and CAA-related inflammation in a 78-year-old man. In addition to the right dominant diffuse white matter lesions, an axial GRE T2*-weighted image on the 1.5-T imager (**a**) revealed some cortical-subcortical hypointense foci suggestive of CAA-related MBs (*arrows*). Of note, more hypointense foci in the posterior dominant distribution were identified on the corresponding PRESTO image (**b**)

The optimal detection of haemorrhagic lesions, including MB and SS, depends on multiple MRI parameters, including pulse sequence, spatial resolution, echo time and field strength. Due to CAA-related pathological changes such as haemosiderin accumulations which lead to large variations in local magnetic fields and a local reduction in T2*, it is necessary to perform the GRE sequence, which is more sensitive to the magnetic susceptibility effect than turbo spin-echo sequences, in the diagnosis of CAA [7]. In the elderly, GRE sequences are essential to check for CAA-related MBs and/or SAH, which can potentially predict life-threatening lobar haemorrhages [8]. Compared with conventional 2D sequences, increasing spatial resolution (i.e. smaller voxel size) on 3D sequence improves the detection of MBs [9]. A longer echo time enables more efficient detection of MBs than a shorter one due to the blooming effect [9]. In addition to these parameters, higher susceptibility effects and increase of signal-to-noise ratio with field strength improve the detection of MBs at a 3-T imager compared with a 1.5-T one [9]. Taking these parameters into consideration, it is reasonable to perform sophisticated 3D sequences with higher spatial resolution and longer echo time including a susceptibility-weighted image (SWI) and the principles of echo-shifting with a train of observations (PRESTO) image to detect MB and SS [9, 10]. Notably, SWI with smaller section thickness and higher magnetic field is currently the most sensitive technique to visualise MBs, which combines both magnitude information and phase information to accentuate the visibility of susceptible foci. These sequences can be of potential value in the evaluation of CAA patients (Fig. 1).

However, the GRE sequence is less sensitive than the FLAIR sequence for the detection of acute SAHs and parenchymal changes. The better lesion/tissue contrast achieved by the suppression of the signal intensity of cerebrospinal fluid (CSF) on the FLAIR sequence not only in the subarachnoid space, but also in the cerebral parenchyma can be especially useful for the evaluation of CAA-related white matter lesions and SAHs. In addition to these sequences, DWI with apparent diffusion coefficient (ADC) maps can be useful to distinguish CAA-related silent infarctions from other white matter lesions, including vasogenic oedema and leukoaraiosis [3, 5].

### PET

Although clinical criteria based on MRI and CT findings have been validated for a pre-mortem diagnosis of CAA during life [11], this relies on detecting late manifestations of CAA-related vascular damage such as ICHs and MBs rather than the vascular amyloid itself. Therefore, these morphological techniques cannot lead to a correct diagnosis in a definite proportion of CAA patients during life. However, an emerging functional technique, Pittsburgh compound B (PiB) PET imaging to measure the burden and location of fibrillar Aβ deposits, has recently been reported as a promising technique for CAA detection [12]. In addition to binding to fibrillar senile plaques, this radiotracer can clearly delineate the vascular amyloid before it triggers haemorrhagic complications or other overt small-vessel brain injuries, and can be a useful clue to diagnose not only AD but also CAA [13].

**Fig. 2:**
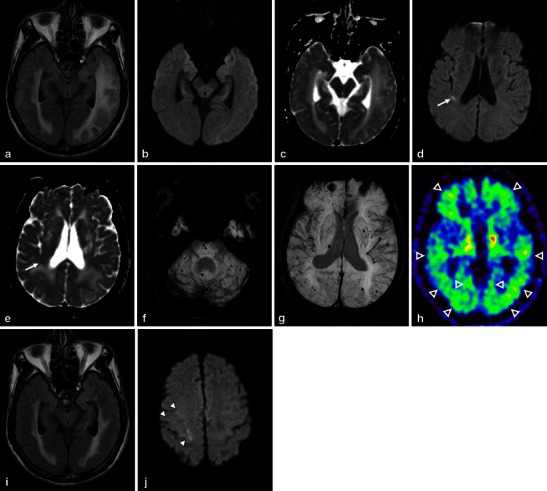
CAA-related inflammation, MBs, and microinfarctions in a 72-year-old man. An axial FLAIR image (**a**) showed large confluent asymmetric hyperintense lesions, which involved not only the left dominant subcortical white matter but also the overlying left temporo-occipital cortices, with a mass effect. Low signal intensity on DWI (**b**) and increased diffusion on the ADC map (**c**) suggested vasogenic oedema. In addition to these white matter lesions, DWI (**d**) demonstrated a small right temporal hyperintense lesion (*arrow*) with corresponding decreased diffusion (*arrow*) on the ADC map (**e**) (*arrow*). This signal change indicated a relatively acute microinfarction. An axial 3D T2*-weighted image (**f**, **g**) revealed multiple MBs, which were distributed not only in the posterior dominant cortical-subcortical region but also in the left putamen, right thalamus, pons and cerebellum. A PiB-PET image (**h**) revealed the diffuse cortical accumulation, including the occipital lobes, higher than those of the cerebral white matter, which indicated the global PiB uptake (*open arrowheads*). These finding of MBs and PiB distribution suggested the coexistence of CAA and hypertensive arteriopathy. Two months after a course of intravenous steroid therapy, an improvement in the white matter lesions was identified on a FLAIR image (**i**). However, DWI (**j**) revealed new subcortical microinfarctions in the right frontal lobe (*arrowheads*)

In the case of coexisting CAA and hypertension, the widespread involvement of arterioles by both types of arteriopathy likely causes the progression of small vessel disease and some overlap in the distribution of MBs [14]. In such cases, PiB-PET can provide a definite clue not only for the diagnosis of CAA but also the coexistence of hypertensive arteriopathy (Fig. 2). However, it is important to understand that PIB is a non-specific imaging marker of Aβ peptide-related cerebral amyloidosis. As previously mentioned, this tracer labels vascular as well as plaque Aβ; therefore, differentiating PiB signal caused by CAA from that caused by other kinds of plaques is difficult.

## Representative imaging findings: multifaceted features of a single pathological condition

### Lobar haemorrhage: a life-threatening sign

CAA is one of the most common causes of lobar ICHs in the elderly [15]. In addition to the advanced age, hypertension and minor head injuries may increase the risk of CAA-related ICHs [15, 16]. The clinical presentation of CAA-related ICH varies according to ICH size and location. Patients commonly present with an acute stroke syndrome with focal neurological deficits that may be associated with headache, vomiting, seizures and/or an altered level of consciousness. In the longer term, survivors of lobar ICHs are at a high risk of recurrence, especially with the presence of the ε2 or ε4 alleles of the apolipoprotein E gene (28 % cumulative recurrence rate at 2 years relative to 10 % in patients without either allele) [17].

**Fig. 3:**
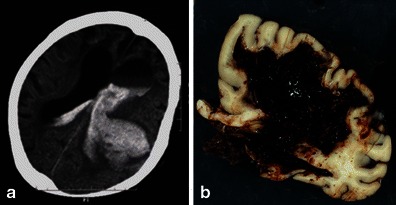
Fetal CAA-related ICH with associated massive ventricular haemorrhage in a 92-year-old woman. A CT scan (**a**) revealed large left-sided parietal subcortical ICH extending into the left lateral ventricle, which caused hydrocephalic ventricular dilatation. A huge subcortical and intraventricular haematoma was identified on a macroscopic specimen at autopsy (**b**)

**Fig. 4:**
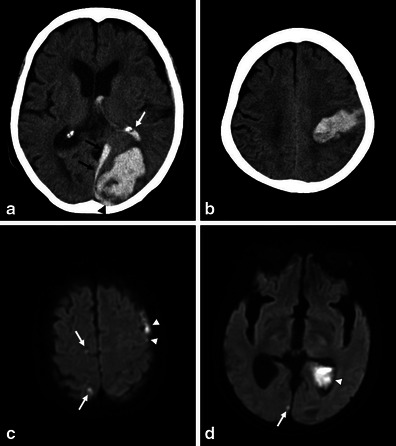
Recurrent CAA-related ICHs associated with intraventricular, subdural and subarachnoid haemorrhages, and microinfarctions in an 87-year-old woman. A CT scan (**a**) showed left-sided occipital subcortical ICH extending into the left lateral ventricle (*white arrow*), and the subdural (*black arrows*) and subarachnoid space (*arrowhead*) around the occipital lobe. Seven months later, left-sided parietal subcortical ICH recurred and extended into the left ventricle. In addition to these haemorrhagic lesions, asymptomatic cortical microinfarctions (*arrows*) were identified on DWI 12 days after the first (**c**) and 13 days after recurrent ICH (**d**). *Arrowheads* indicated subdural, subarachnoid and intraventricular haemorrhages

Regardless of the size, CAA-related ICHs exhibit distinctive cortical-subcortical distributions that generally spare the deep white matter, basal ganglia and brainstem. This cortical-subcortical distribution of CAA-related ICHs has been correlated with the anatomical distribution of β-amyloid-containing vessels. Notably, haemorrhagic lesions are shown to be preferentially distributed in the temporal and occipital lobes, and are likely to cluster regardless of the lobes [18]. Comprehension of this characteristic distribution was validated by the Boston criteria, which are most commonly used and highly specific for the diagnosis of CAA (Table 3) [11, 19]. CAA-related macrohaemorrhages may be associated with subarachnoid, subdural or, less commonly, intraventricular haemorrhage (Figs. 3 and 4) [8]. Other neuroimaging findings suspicious for CAA-related ICHs include the multiplicity and recurrence of ICHs (Fig. 4). Recurrent haemorrhages are typically lobar, often in the same lobe as the initial CAA-related ICHs [18]. CT is sufficient to provide crucial information regarding the characteristics of acute CAA-related ICHs. However, MRI examinations including GRE sequences should be performed to evaluate chronic haemosiderin depositions and MBs, which can be useful to diagnose CAA.

**Table 3:** Classic and modified Boston criteria [11, 19]

	Classic Boston criteria	Modified Boston criteria
Definite CAA	Full post-mortem examination demonstrating:	No modification
	- Lobar, cortical or corticosubcortical haemorrhage	
	- Severe CAA with vasculopathy	
	- Absence of other diagnostic lesion	
Probable CAA with supporting pathology	Clinical data and pathological tissue (evaluated) haematoma or cortical biopsy) demonstrating:	No modification
	- Lobar, cortical or corticosubcortical haemorrhage	
	- Some degree of CAA in specimen	
	- Absence of other diagnostic lesion	
Probable CAA	Clinical data and MRI or CT demonstrating:	Clinical data and MRI or CT demonstrating:
	- Multiple haemorrhages restricted to lobar, cortical or corticosubcortical regions (cerebellar haemorrhage allowed)	- Multiple haemorrhages restricted to lobar, cortical or corticosubcortical regions (cerebellar haemorrhage allowed), or
	- Age ≥55	- Single lobar, cortical, or corticosubcortical haemorrhage and focal or disseminated superficial siderosis
	- Absence of other cause of haemorrhage	- Age ≥55
		- Absence of other cause of haemorrhage or superficial siderosis
Possible CAA	Clinical data and MRI or CT demonstrating:	Clinical data and MRI or CT demonstrating:
	- Single lobar, cortical or corticosubcortical haemorrhage	- Single lobar, cortical or corticosubcortical haemorrhage, or
	- Age ≥55	- Focal or disseminated superficial siderosis
	- Absence of other cause of haemorrhage	- Age ≥55
		- Absence of other cause of haemorrhage or superficial siderosis

### Microbleeds: easily overlooked but a suggestive sign of CAA

**Fig. 5:**
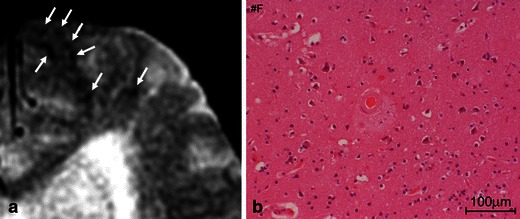
Pathologically proved subcortical MBs in a 76-year-old man. An axial GRE T2*-weighted image (**a**) showed multiple cortical-subcortical hypointense foci suggestive of CAA-related MBs (*arrows*). A histopathological section corresponding to MBs (haematoxylin and eosin stain) (**b**) revealed amyloid deposits in the vessel walls with perivascular leakage of erythrocytes and plasma

This finding indicates previous extravasation of blood-related to bleeding-prone microangiopathy, including CAA and hypertensive arteriopathy (Fig. 5) [14]. In the pathological analysis of lobar MBs in CAA patients, various CAA-related pathologies, including acute microhaemorrhages, haemosiderin residua of old haemorrhages and small lacunes ringed by haemosiderin, are proved to produce signal voids on SWI [20]. MBs located in lobar regions may correlate with disease progression, recurrent ICH, and cognitive dysfunctions [21, 22]. Moreover, early recognition can be advantageous to patients on antiplatelet or thrombolysis therapy in that they are at an increased risk for subsequent and possibly fatal haemorrhages [4, 6].

**Fig. 6:**
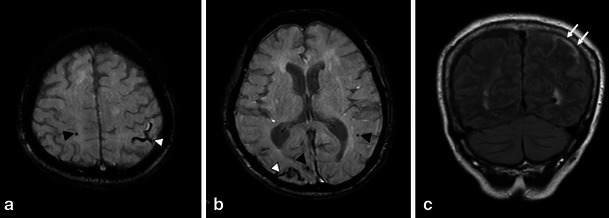
Non-traumatic SAH at the convexity of the brain, microbleed and SS in a 72-year-old woman who was clinically diagnosed with AD. Axial 3D T2*-weighted images (**a**, **b**) showed bilateral subcortical MBs (*black arrowheads*) and sulcal SS (*white arrowheads*) in the posterior dominant distribution. Additionally, SAH along the left parietal sulci (*arrows*) was identified on a coronal FLAIR image (**c**)

**Fig. 7:**
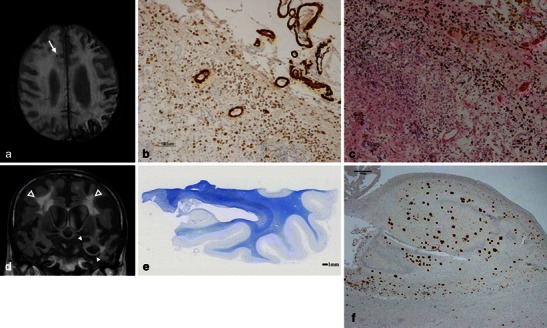
CAA-related SS and MB in a 90-year-old man with pathologically proved AD. In addition to the right frontal subcortical MB (*arrow*), a 3D T2*-weighted image (**a**) demonstrated the typical gyriform low signals along the left cerebral sulci. Severe amyloid beta immunoreactive deposits were present in the leptomeningeal and cortical vessel walls of the parietal lobe (immunohistochemistry raised against monoclonal antibody Aβ 11–28) (**b**). The upper cortical layers were necrotic. Numerous haemosiderin-laden macrophages were present in the subarachnoid space and upper cortical layers (haematoxylin and eosin stain). These findings were consistent with superficial siderosis. A coronal FLAIR image (**d**) demonstrated left dominant atrophy of the amygdala and parahippocampal gyrus (*arrowheads*), and symmetric deep white matter hyperintensities (*open arrowheads*). A section of the left posterior hippocampus revealed atrophy of the hippocampus proper, subiculum and parahippocampal gyrus. Pallor of the subcortical white matter was evident (Klüver-Barrera stain) (**e**). There were numerous Aβ 11–28 immunoreactive senile plaques in the hippocampus (**f**)

MBs are typically defined on GRE sequences as small, well-demarcated, hypointense, rounded foci less than 5–10 mm in size, which are distinct from cortical vascular flow voids, leptomeningeal siderosis, or non-haemorrhagic subcortical mineralisation such as symmetrical hypointensities in the globus pallidus [21]. The presence of multiple, strictly lobar, cortical-subcortical MBs detected by GRE sequences has been shown to be highly specific for severe CAA in elderly patients with no other definite cause of ICH, such as trauma, ischaemic stroke, coagulopathy or excessive anticoagulation (probable CAA on the Boston criteria in the Table 3) [11]. Similar to the distribution of CAA pathology and CAA-related lobar ICHs, the distribution of CAA-related MBs appears to show a posterior cortical predominance (Figs. 1, 2 and 6) [18]. GRE sequences are the recommended method for MB detection due to the insensitivity of MB detection on CT and spin-echo sequences of MRI. Furthermore, considering the limitation of conventional T2* GRE sequences, which have underestimated MBs in 25 % of CAA patients, more sensitive sequences such as SWI and PRESTO sequences should be used to increase the detection rates of MBs (Fig. 1) [9]. It is notable that neuroimaging study has revealed lobar MBs in more than 20 % of patients with AD (Fig. 7), which may reflect advanced CAA in keeping with neuropathological findings [23].

### Subarachnoid haemorrhage: a predictive finding of unfavourable outcomes?

Recently, CAA has been increasingly reported as a cause of SAHs in the elderly, especially those localised at the convexity of the brain (cSAH) [24, 25]. CAA-related cSAH may be due to direct extension of the cortical-subcortical haemorrhage into the subarachnoid or to primary SAH resulting from disruption of the leptomeningeal vessels by β-amyloid (Figs. 4 and 6) [8]. The clinical presentation of CAA-related cSAH is distinct because patients suffer from transient focal neurological deficits, including motor or sensory symptoms and seizures, rather than typical headaches [24, 25]. Such symptomatic cSAHs are mainly located within the central sulcus. Whether cSAH could be a warning sign of subsequent ICHs depends on the underlying disease. CAA-related cSAH often recurs, and a high rate of subsequent cerebrovascular disorders including infarctions and ICHs could contribute to unfavourable outcomes, including neurological disability and death in the elderly [25, 26].

Unenhanced head CT has shown a slight, sometimes barely visible, sulcal hyperattenuation, most frequently depicted around the precentral gyrus [26]. Subsequent MRI scans confirmed the subarachnoid haemorrhage as a hyperintense area on FLAIR images (Fig. 6). In addition to this subarachnoid lesion, GRE sequences, especially SWI and PRESTO images, showed multiple lobar cortical-subcortical haemorrhagic lesions (macrohaemorrhages or MBs) (Fig. 6) [24, 25]. Considering the high prevalence of MBs and SS in CAA patients [24], these abnormal findings should be evaluated in the diagnosis of cSAH. It is also to be noted that CAA-related cSAH and SS can be present without other haemorrhagic lesions, including ICHs and MBs [19].

### Superficial siderosis: a clinical entity distinct from the well-known classical SS

It is estimated that repeated cSAH leads to haemosiderin deposits in the subpial layers of the supratentorial brain [19]. In addition to other lobar haemorrhagic lesions, SS depicted predominantly in the supratentorial area has been increasingly recognised as one of the CAA-related abnormal findings [24]. A recent report has revealed that CAA-related SS as well as cSAH can be a warning sign of future intracranial haemorrhagic lesions [27].

Considering the marked difference of SS prevalence observed between CAA and non-CAA patients, the inclusion of SS in the modified Boston criteria may enhance their sensitivity for the diagnosis of CAA without a loss in specificity (Table 3) [19]. Furthermore, SS can be the important indicator of CAA in AD patients beyond the MBs or ICHs that are more readily recognised as being CAA-related haemorrhagic lesions (Fig. 7) [28].

CAA-related SS on GRE sequences showed the characteristic ‘gyriform’ pattern of a hypointense signal (Figs. 6 and 7). Generally, proton density and FLAIR images or unenhanced CT scans are used to identify acute SAHs and to distinguish them from chronic SS [27]. This abnormal signal intensity revealed a preference for the cerebral convexity and only exceptionally occurred in the infratentorial area [19]. This distribution explains why CAA-related SS may be associated with transient neurological manifestations and lacks the typical clinical presentation associated with the well-known SS, namely cerebellar and brainstem signs [19].

### CAA-related inflammation—treatable form of a CAA-related disorder

In addition to haemorrhagic complications, a syndrome of perivascular inflammation and oedema has been recognised in the spectrum of presentations associated with CAA [29]. Pathologically, CAA-related inflammation reveals vascular amyloid deposition accompanied by perivascular, intramural and/or transmural inflammatory changes, with or without granuloma formation. The mechanism by which this immune response occurs is not well understood, although one possible factor is the increased frequency of the apolipoprotein E ε4/ε4 genotype [5, 29]. The clinical presentation of CAA-related inflammation typically manifests as headache, subacute cognitive decline and seizures [5, 29]. The apparent response of most patients to immunosuppressive therapy suggests that this disorder represents a treatable form of CAA, which highlights the importance of reaching this diagnosis in practice (Fig. 2). However, CAA-related inflammation could also be not only a stable/progressive disorder but also a relapsing disorder, and the proportion crossing over from “improved” to “relapsing” disease may increase with longer follow-ups [5].

CAA-related inflammation is characterised by large confluent asymmetric white matter lesions of abnormal attenuation/intensity extending to the subcortical white matter and occasionally the overlying cortical grey matter with mass effect (Figs. 1 and 2). These lesions are depicted more clearly on MRI than on CT, especially on the FLAIR sequence, and involve one or more cortical territories, distributed almost equally across the frontal, parietal, temporal and occipital lobes without evident preferential laterality [5]. DWI and ADC maps can add further information suggestive of vasogenic oedema (Fig. 2). Interestingly, the clinical and neuroimaging feature of this condition is similar to the vasogenic oedema of amyloid-related imaging abnormalities (ARIA) associated with amyloid-modifying therapy. A potential connection between CAA-related inflammation and immunotherapy-associated ARIA has been recently suggested by identification of anti-Aβ autoantibodies in the CSF of a patient with the spontaneously occurring syndrome [30].

### Leukoaraiosis: a common but by no means specific finding of CAA

Leukoaraiosis is a radiological term which describes the abnormal imaging changes in the deep cerebral white matter. Pathological changes include demyelination, axon loss and mild gliosis. CAA-related impairments of perfusion due to amyloid in the overlying cortical small vessels probably cause the leukoaraiosis in CAA patients [3, 32]. Another possible mechanism of leukoaraiosis in CAA is as a result of the accumulation of silent ischaemic lesions [3].

**Fig. 8:**
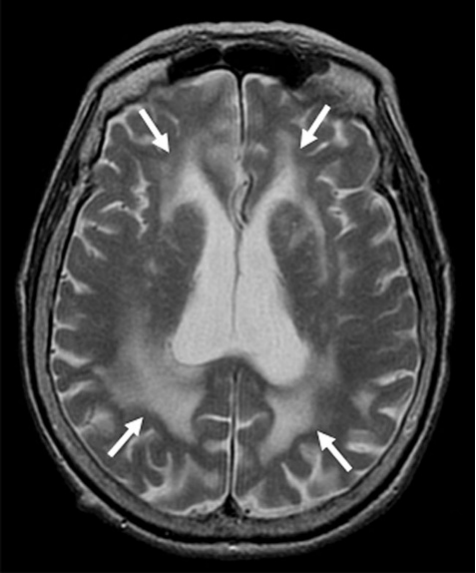
Leukoaraiosis in an 87-year-old woman with pathologically proved CAA and AD. Axial T2-weighted images showed bilateral hyperintensities (*arrows*), which involved the posterior dominant periventricular and deep white matters

Leukoaraiosis appears as diffuse or focal low attenuation on CT or hyperintensity on T2-weighted and FLAIR images on MRI, which is prevalent in the centrum semiovale and deep white matter with sparing of the subcortical U fibres (Figs. 7 and 8) [8]. In contrast to CAA-related inflammation, this finding is irreversible. As well as hypertensive arteriopathy, CAA-related leukoaraiosis preferentially affects the same periventricular regions; however, some studies suggest the posterior dominant white matter involvement in CAA patients [3, 32] (Fig. 8). Although advanced CAA is associated with a large burden of white matter lesions compared with healthy elders and AD patients, these lesions are basically non-specific and not useful for the diagnosis of CAA.

### Microinfarction: a clinically silent event suggestive of progressive arteriopathy

Recent studies using MR DWI, which is very sensitive to even small ischaemic lesions, have demonstrated that acute and subacute ischaemic infarctions are not infrequent in patients with advanced CAA, and occur in approximately 15 % of these patients [31, 32]. Analyses of autopsied brains with advanced CAA have identified lesions described as perivascular scars or small infarctions at frequencies ranging from 37 % to nearly 100 % [32]. These pathologically observed infarctions are frequently multiple and located in the cortical ribbon or underlying subcortical white matter. Impaired cerebral blood flow regulation due to CAA-related occlusive arteriopathy may be related to these ischaemic changes [32]. Their presence was shown to be unrelated to conventional vascular risk factors such as hypertension, diabetes and coronary artery disease, and was instead associated with the severity of white matter lesions and lobar MBs, which suggests that they are due to CAA-related occlusive arteriopathy [31]. These lesions appear to be clinically asymptomatic; however, the therapeutic implications and prognostic significance of these findings require further study.

On MRI, these lesions are located mainly in the subcortical white matter and cortical grey matter away from the site of previous ICHs [31, 32]. They may also be located in the cerebellum. Acute lesions were identified as small and mostly ovoid or round bright areas on DWI sequences and corresponding dark areas on ADC maps (Figs. 2 and 4).

### CAA and AD—representation of two sides of a single condition: Aβ amyloidosis

Pathologically, CAA is commonly found in AD (Fig. 7), with a prevalence of more than 80 % [2]. The high prevalence of CAA in patients with AD as well as of cerebral parenchymal Aβ deposition (senile plaques) in patients with CAA can be explained by AD and CAA representing two sides of a single condition. Therefore, the degree of CAA in AD is more severe than that in non-AD patients. It is noteworthy that the presence of CAA may have a significant impact on the clinical course of AD. The coexistence of CAA with AD has been reported to impair cognitive performance more significantly than AD alone, even after adjustments for age, neurofibrillary tangle and amyloid plaque number, and infarctions [33].

## Differential diagnosis

A single large cortical-subcortical ICH presenting with an acute neurological deficit is not entirely specific for a diagnosis of CAA. Various disorders, including hypertensive arteriopathy, haemorrhagic tumours, vascular malformation, trauma, bleeding diatheses and illicit drug use such as amphetamines and cocaine, can cause cortical-subcortical ICHs [8]. Notably, infectious aneurysm can cause not only subcortical ICH but also SAH and MBs like signal change. In the diagnosis of subcortical haemorrhagic lesions, it is sometimes difficult to narrow the differential diagnosis because of its non-specific nature. Therefore, to evaluate other abnormal findings suggestive of CAA (i.e. MBs and SS) is mandatory for the precise diagnosis. Gadolinium enhancement and MR angiogram are also useful to evaluate the tumorous and vascular lesions, respectively.

The hypertensive arteriopathy as well as CAA is the most common cause of MBs. Less common causes include diffuse axonal injury, cerebral fat embolism, cerebral autosomal dominant arteriopathy with subcortical infarcts and leukoencephalopathy (CADASIL), multiple cavernous malformations, vasculitis, radiation vasculopathy and so on. To understand the distinctive cortical-subcortical distributions that generally spare the basal ganglia and brainstem is important for the diagnosis of CAA. To check the other imaging findings such as restricted diffusion of axonal injury, multiple white matter lesions—especially in the temporal pole of CADASIL—and vascular lesions of vasculitis, are also contributory to diagnosis.

Convexal SAH and SS are important subtypes of nonaneurysmal subarachnoid bleeding and its sequela with diverse aetiologies. Although CAA is frequent in patients older than 60 years, a reversible vasoconstriction syndrome appears to be a common cause of cSAH in younger patients [24]. Other than those above, cSAH carries a broad differential diagnosis, including head trauma, posterior reversible encephalopathy syndrome (PRES), dural sinus and cortical venous thrombosis, vascular malformation, vasculitis and anticoagulation [34]. Parenchymal abnormalities, including cerebral contusions and subcortical white matter lesions, are useful to diagnose head trauma and PRES. Additionally, to check the vascular lesion, especially the dural sinus and cortical vein, is crucial for the diagnosis of thromboses and malformations.

Because various pathological conditions, including PRES, infections (e.g. progressive multifocal leukoencephalopathy), acute disseminated encephalomyelitis and malignancies (e.g. primary CNS lymphoma and gliomatosis cerebri), can manifest as multiple white matter lesions [35], the essential step in the diagnosis of CAA-related inflammation is the recognition of CAA. In other words, GRE sequences including SWI and PRESTO images, which enable recognition of CAA-related MBs and SS, are fundamental in diagnosing CAA-related inflammation without invasive brain biopsy (Fig. 2) [5]. Considering that a part of the CAA-related inflammation may manifest as non-haemorrhagic white matter lesions, PiB-PET should be regarded as a supplementary diagnostic technique.

In addition to above-described imaging findings, it is also necessary to evaluate the medical history, physical examination findings and laboratory results to differentiate CAA from its mimickers. For example, typical medical history such as the elevated blood pressure and chemotherapy is usually associated with PRES and helps to clarify the diagnosis.

## Conclusions

In the various types of CAA-related abnormal findings, haemorrhagic lesions, especially lobar restricted ICHs and MBs, cSAH and supratentorial SS, in the elderly can be crucial imaging findings of CAA. Furthermore, CAA can reveal other imaging findings including CAA-related inflammation and microinfarction. Radiologists should understand that MRI, especially GRE and FLAIR sequences, can non-invasively provide clues for the diagnosis of CAA-related disorders. CAA-related imaging findings are not always specific; therefore, it is necessary to combine with other CAA-related imaging findings for the diagnosis. Amyloid PET can be an important clue to differentiate CAA from other pathological conditions, such as hypertension and bleeding diatheses, which cause similar and mistakable haemorrhagic imaging findings.
